# Supplementary material for: Acceptance of a Text Messaging Vaccination Reminder and Recall System in Malaysia’s Healthcare Sector: Extending the Technology Acceptance Model
Source: Vaccines (Basel). 2023 Aug 6;11(8):1331. doi: 10.3390/vaccines11081331 (PMC10458098; doi:10.3390/vaccines11081331)
Supplement: Supplementary file 1 [file vaccines-11-01331-s001.zip › vaccines-2490569-supplementary.pdf]

Supplementary

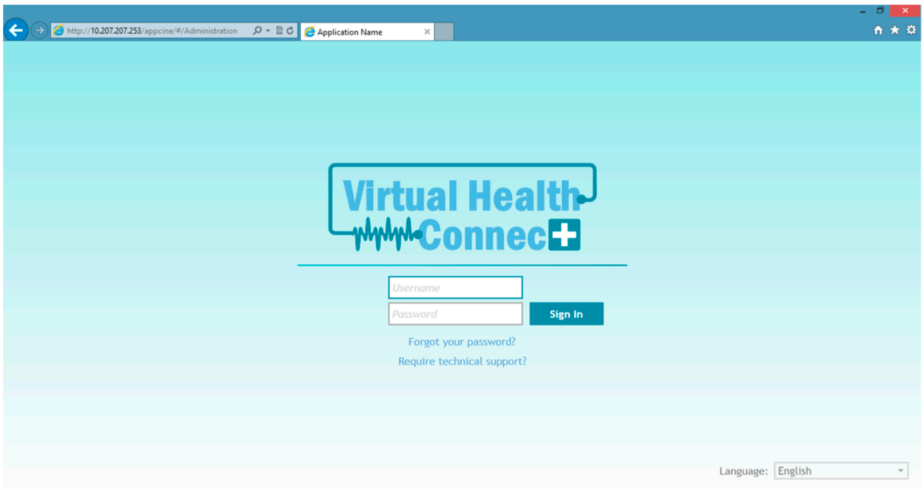

(a) Login Page

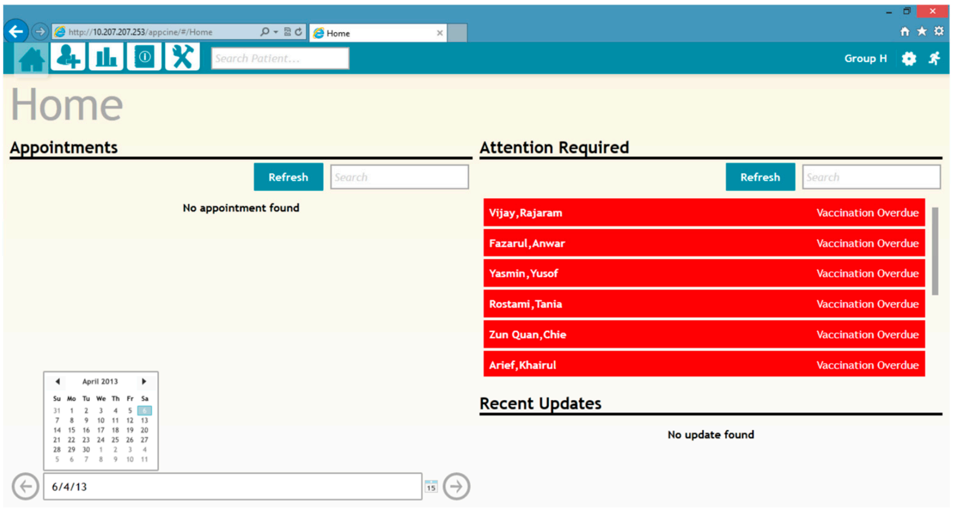

(b) Dashboard

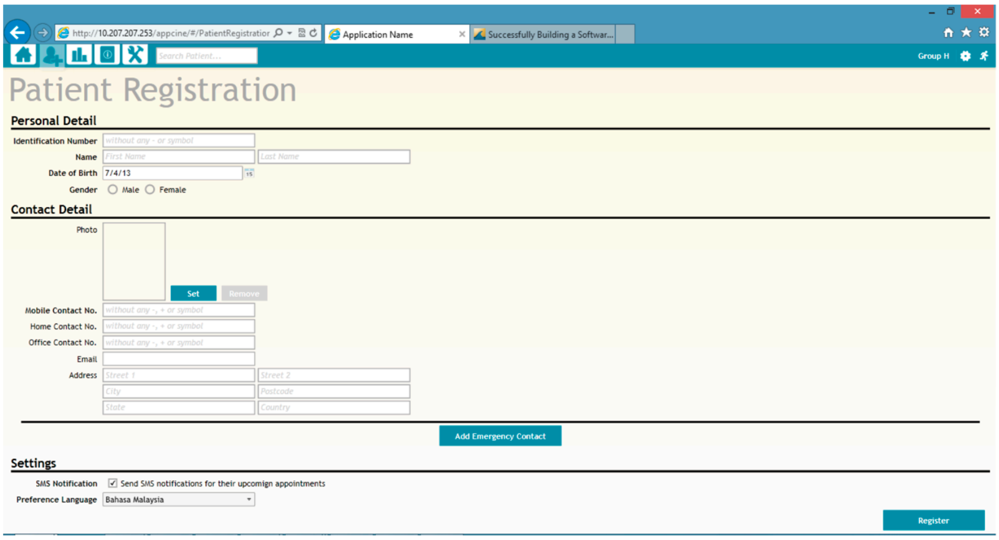

(c) Patient (Child) Registration

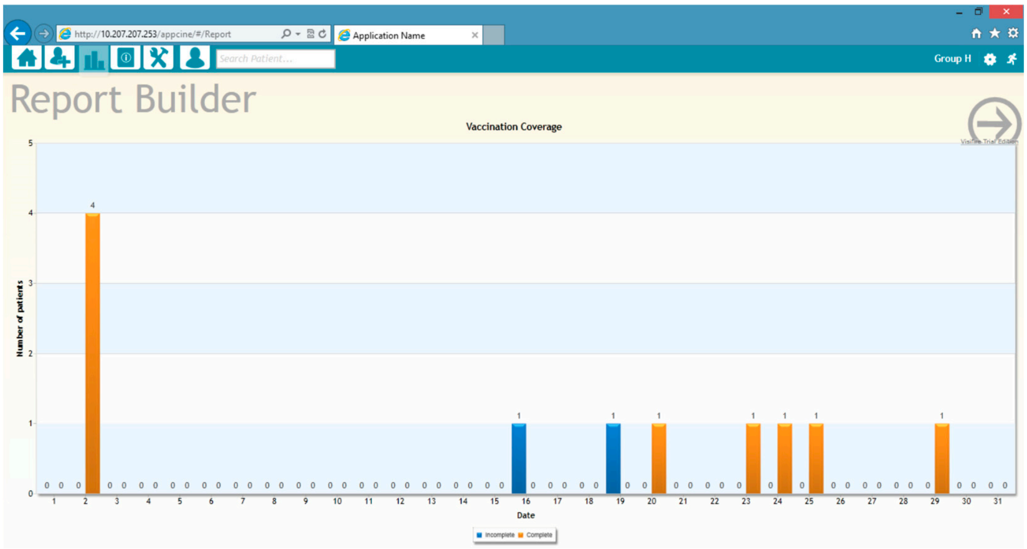

(d) Vaccination Report

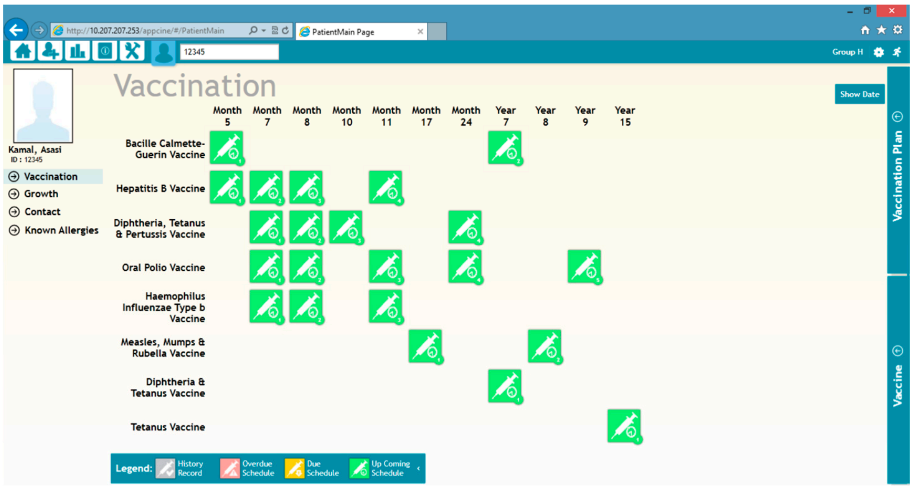

(e) Applied Vaccination Plan

Figure S1. The Snapshots of VHC: (a) Login Page, (b) Dashboard, (c) Patient (Child) Registration, (d) Vaccination Report, and (e) Applied Vaccination Plan.
